# Effectiveness of SGLT2 Inhibitors in Improving Functional Capacity and Reducing Hospitalizations in Heart Failure With Preserved Ejection Fraction (HFpEF): A Systematic Review of Randomized Controlled Trials

**DOI:** 10.7759/cureus.92372

**Published:** 2025-09-15

**Authors:** Sangeen Khan, Ayesha Ashraf, Mansoor Awais, Mussavir Elahi, Mahrukh Chaudhry, Allahyar Khar

**Affiliations:** 1 General Medicine, Fairfield General Hospital, Bury, GBR; 2 Internal Medicine, King Edward Medical University, Lahore, PAK; 3 Internal Medicine, Nishtar Medical University, Multan, PAK

**Keywords:** cardiovascular outcomes, dapagliflozin, empagliflozin, functional capacity, heart failure hospitalization, hfpef, kccq, quality of life, randomized controlled trials, sglt2 inhibitors

## Abstract

Sodium-glucose co-transporter 2 (SGLT2) inhibitors have recently emerged as a promising therapeutic option for patients with heart failure with preserved ejection fraction (HFpEF), a condition historically resistant to pharmacological interventions. This systematic review synthesizes evidence from 10 randomized controlled trials evaluating the effects of empagliflozin and dapagliflozin on functional capacity and hospitalization outcomes in HFpEF patients. The findings consistently demonstrate significant improvements in patient-reported outcomes, such as the Kansas City Cardiomyopathy Questionnaire (KCCQ) scores and six-minute walk test (6MWT) performance, along with reductions in heart failure hospitalizations. These benefits were observed across diverse patient populations, including older adults and those with comorbidities like diabetes and chronic obstructive pulmonary disease, with a favorable safety profile. The review supports the incorporation of SGLT2 inhibitors as a central component in the management of HFpEF, offering both symptomatic relief and a reduction in clinical events.

## Introduction and background

Heart failure with preserved ejection fraction (HFpEF) accounts for nearly half of all heart failure cases worldwide and presents a growing clinical challenge, particularly among older adults and patients with comorbidities such as hypertension, obesity, diabetes, and atrial fibrillation [1]. Unlike heart failure with reduced ejection fraction (HFrEF), therapeutic strategies for HFpEF have historically yielded limited success, with most pharmacological agents failing to show consistent improvements in morbidity and mortality. HFpEF is characterized by symptoms of heart failure - such as dyspnea, fatigue, and exercise intolerance - despite a preserved left ventricular ejection fraction (LVEF ≥50%) [2]. These symptoms contribute significantly to reduced functional capacity and frequent hospitalizations, ultimately impairing quality of life and increasing healthcare burdens. In addition to the clinical challenges, HFpEF imposes a substantial economic burden on healthcare systems due to repeated admissions, costly diagnostic evaluations, and ineffective therapeutic strategies that have not translated into survival benefit. Several pharmacological classes, including renin-angiotensin-aldosterone system (RAAS) inhibitors, beta-blockers, and neprilysin inhibitors, have shown limited or inconsistent efficacy in improving outcomes for this population, highlighting the urgent need for more effective options.

In recent years, sodium-glucose co-transporter 2 (SGLT2) inhibitors - initially developed as antidiabetic agents - have demonstrated compelling cardiovascular and renal benefits beyond glycemic control [3]. Large-scale randomized controlled trials (RCTs) have evaluated the efficacy of agents such as empagliflozin and dapagliflozin in patients with HFpEF. These trials have not only suggested a reduction in heart failure hospitalizations but also improvements in functional outcomes as measured by tools like the Kansas City Cardiomyopathy Questionnaire (KCCQ) [4], 6-minute walk test (6MWT) [5], and quality of life metrics [6].

Despite these encouraging results, the extent to which SGLT2 inhibitors improve functional capacity and reduce hospitalization rates in HFpEF patients remains an area requiring focused synthesis. Existing literature includes various subgroup analyses and post-hoc studies that examine differential effects by age, sex, comorbidity status, and other clinical variables, contributing to a complex and evolving evidence base. This systematic review aims to evaluate the effectiveness of SGLT2 inhibitors - specifically empagliflozin and dapagliflozin - in improving functional capacity and reducing hospitalization in patients with HFpEF. By synthesizing evidence from randomized controlled trials and high-quality clinical studies, the review seeks to clarify their clinical utility and inform future therapeutic strategies for this difficult-to-treat population.

## Review

Materials and methods

Study Design and Objectives

This study was conducted as a systematic review of randomized controlled trials (RCTs), adhering to the Preferred Reporting Items for Systematic Reviews and Meta-Analyses (PRISMA) guidelines [7]. The review followed the PICO framework (Population, Intervention, Comparator, Outcomes) [8], defining the Population as patients with heart failure with preserved ejection fraction (HFpEF, consistently defined as LVEF ≥50%), the Intervention as sodium-glucose co-transporter 2 (SGLT2) inhibitors (specifically empagliflozin and dapagliflozin), the Comparator as placebo or standard care, and the Outcomes as changes in functional capacity and clinical endpoints. The primary objective was to assess the impact of these agents on functional outcomes, such as the Kansas City Cardiomyopathy Questionnaire (KCCQ) and six-minute walk test (6MWT), as well as on cardiovascular mortality and heart failure-related hospitalizations. The secondary objective was to explore patterns of efficacy across subgroups defined by age, baseline functional status, and comorbid conditions.

Given the heterogeneity in trial designs, populations, outcome measures, and follow-up durations, a quantitative meta-analysis was not performed because a high degree of statistical inconsistency (I²) was anticipated. Instead, a qualitative synthesis was undertaken to capture clinically relevant patterns while minimizing the risk of misleading pooled estimates. The search was limited to four major databases (PubMed, MEDLINE, Embase, and the Cochrane Central Register of Controlled Trials), which may not fully exclude publication bias despite comprehensive efforts.

Eligibility Criteria

Studies were included if they met the following criteria: randomized controlled trial design; adult patients with HFpEF, defined as left ventricular ejection fraction (LVEF) >40% or ≥50%, depending on trial-specific definitions; evaluation of empagliflozin or dapagliflozin as the intervention; comparison with placebo or standard care; and reporting of at least one of the following outcomes - functional capacity (KCCQ, 6MWT), quality of life, heart failure hospitalization, or cardiovascular mortality. Studies were excluded if they were non-randomized, focused exclusively on heart failure with reduced ejection fraction (HFrEF), or involved combination therapies without stratified data for SGLT2 inhibitors.

Search Strategy and Data Sources

A comprehensive search of PubMed, MEDLINE, Embase, and the Cochrane Central Register of Controlled Trials was conducted to identify eligible studies published between January 2018 and May 2024. The search strategy combined terms related to HFpEF, sodium-glucose co-transporter 2 (SGLT2) inhibitors, and study design using Boolean operators. A representative PubMed search string was: (“heart failure with preserved ejection fraction” OR “HFpEF” OR “diastolic heart failure”) AND (“sodium-glucose cotransporter 2 inhibitors” OR “SGLT2 inhibitors” OR “empagliflozin” OR “dapagliflozin”) AND (“randomized controlled trial” OR “RCT” OR “clinical trial”). Equivalent search terms and operators were adapted for MEDLINE, Embase, and the Cochrane Central Register of Controlled Trials to ensure comprehensive coverage. In addition, reference lists of retrieved articles were manually screened to identify further eligible studies. Only studies published in English and involving human participants were considered.

Data Extraction and Synthesis

Two independent reviewers screened titles and abstracts for eligibility and subsequently reviewed the full texts of selected studies. Discrepancies were resolved through discussion and consensus with a third reviewer. Data extracted included study design, sample size, population characteristics, intervention and comparator details, follow-up duration, and reported outcomes. Outcomes were synthesized qualitatively, focusing on changes in functional capacity scores (KCCQ, 6MWT), heart failure hospitalization rates, and cardiovascular mortality. The findings were narratively summarized, with emphasis on patterns across age groups, baseline health status, and comorbidities.

Risk of Bias Assessment

The risk of bias for each included study was independently evaluated by two reviewers using the Cochrane Risk of Bias 2.0 tool [9]. Domains assessed included the randomization process, deviations from intended interventions, completeness of outcome data, measurement of outcomes, and selection of reported results. Most studies were determined to have a low risk of bias across all domains, with the exception of one trial, which had some concerns related to sample size and randomization transparency.

Results

Study Selection Process

**Figure 1:**
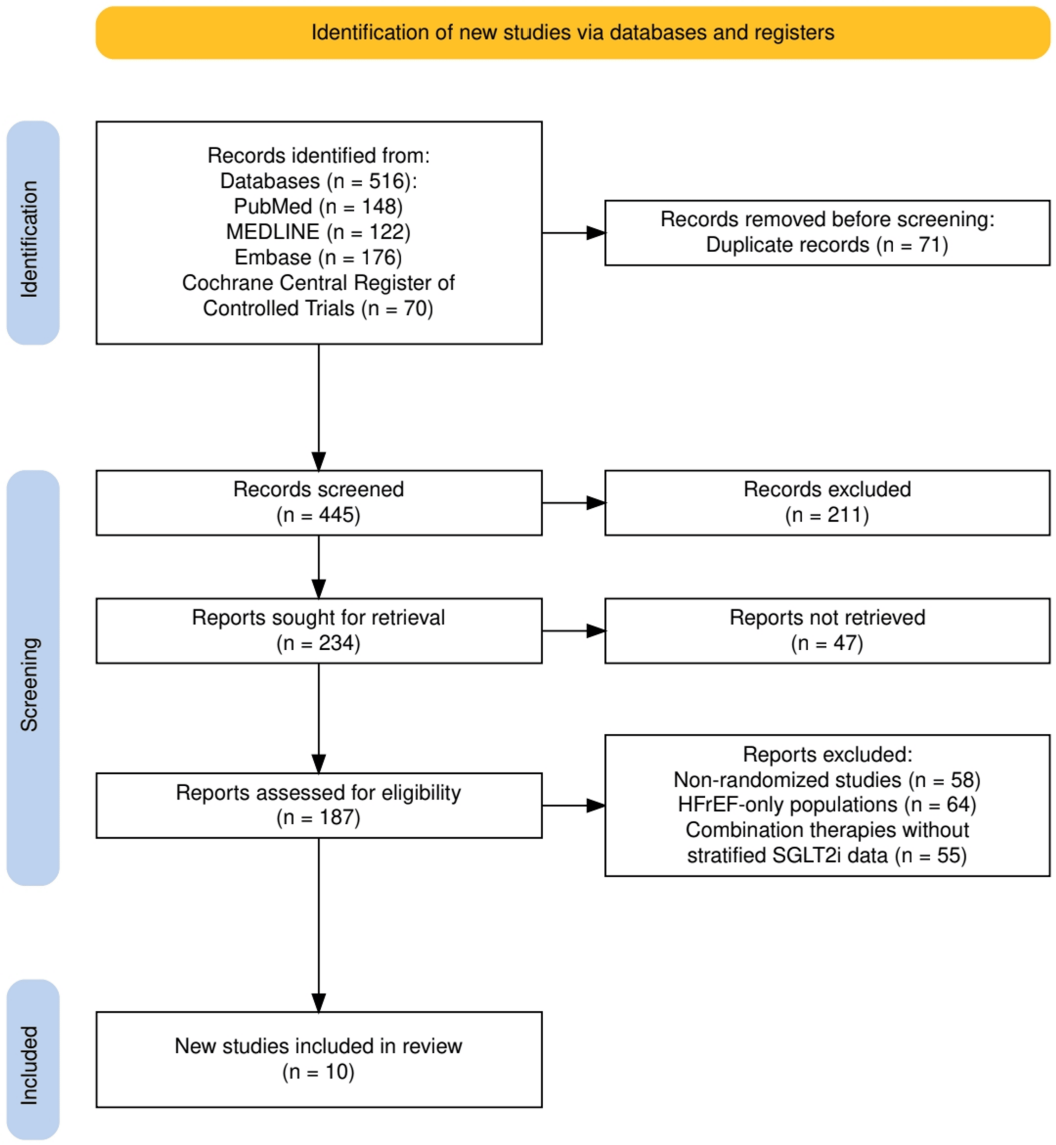
The PRISMA flow diagram represents the study selection process. PRISMA: Preferred Reporting Items for Systematic Reviews and Meta-Analyses

A comprehensive literature search identified a total of 516 records from four major databases: PubMed (n = 148), MEDLINE (n = 122), Embase (n = 176), and the Cochrane Central Register of Controlled Trials (n = 70). After removing 71 duplicates, 445 unique records were screened based on title and abstract. Of these, 211 were excluded for not meeting the eligibility criteria. The full texts of the remaining 234 records were sought for further assessment, but 47 could not be retrieved. Subsequently, 187 full-text articles were reviewed for eligibility. A total of 177 studies were excluded due to reasons such as non-randomized study design (n = 58), exclusive focus on heart failure with reduced ejection fraction (HFrEF) (n = 64), and evaluation of combination therapies without stratified SGLT2 inhibitor data (n = 55). Ultimately, ten randomized controlled trials were included in this systematic review. The complete study selection process is illustrated in Figure 1 (PRISMA flow diagram).

Characteristics of the Selected Studies

Table 1 summarizes the key characteristics and findings of multiple randomized controlled trials investigating the effects of SGLT2 inhibitors in patients with heart failure with preserved ejection fraction (HFpEF). Across a wide range of study populations - varying in age, comorbidities, and baseline functional status - both dapagliflozin and empagliflozin consistently demonstrated improvements in clinical and patient-reported outcomes. These included significant reductions in cardiovascular death, heart failure hospitalizations, and improvements in health-related quality of life measures such as the Kansas City Cardiomyopathy Questionnaire (KCCQ) and EQ-5D-5L scores. Some studies also reported enhanced functional capacity, weight reduction, and favorable hemodynamic effects like decreased pulmonary capillary wedge pressure. Benefits were observed across different subgroups, including older adults, patients with chronic obstructive pulmonary disease (COPD), and those with varying degrees of baseline symptom burden. Importantly, the tolerability of these interventions was comparable to placebo, with no significant increase in adverse events, supporting their safety and effectiveness in a broad HFpEF population.

**Table 1:** The summary of the included studies in the review. HFpEF – Heart Failure with Preserved Ejection Fraction HFrEF – Heart Failure with Reduced Ejection Fraction HFmrEF – Heart Failure with Mildly Reduced Ejection Fraction NYHA – New York Heart Association (functional class) LVEF – Left Ventricular Ejection Fraction EF – Ejection Fraction KCCQ – Kansas City Cardiomyopathy Questionnaire KCCQ-CS / KCCQ-CSS – Kansas City Cardiomyopathy Questionnaire Clinical Summary Score KCCQ-OS – Kansas City Cardiomyopathy Questionnaire Overall Summary Score KCCQ-TS – Kansas City Cardiomyopathy Questionnaire Total Symptom Score 6MWT – Six-Minute Walk Test NT-proBNP – N-terminal pro–B-type Natriuretic Peptide HbA1c – Hemoglobin A1c SBP – Systolic Blood Pressure PCWP – Pulmonary Capillary Wedge Pressure VAS – Visual Analogue Scale eGFR – Estimated Glomerular Filtration Rate HR – Hazard Ratio CI – Confidence Interval COPD – Chronic Obstructive Pulmonary Disease HFH – Heart Failure Hospitalization DAPA-HF – Dapagliflozin and Prevention of Adverse Outcomes in Heart Failure trial DELIVER – Dapagliflozin Evaluation to Improve the Lives of Patients With Preserved Ejection Fraction Heart Failure trial EQ-5D-5L – EuroQol 5 Dimensions, 5 Levels

Author(s)	Study Design	Population Characteristics	Intervention	Comparator	Outcomes Measured	Duration of Follow-Up	Main Findings
Nassif et al. 2021 [10]	Multicenter Randomized Controlled Trial	324 patients with HFpEF (preserved ejection fraction; NYHA class II-IV)	Dapagliflozin	Placebo	Primary: KCCQ Clinical Summary Score (KCCQ-CS); Secondary: 6-minute walk test (6MWT), KCCQ Overall Summary (KCCQ-OS), weight, NT-proBNP, HbA1c, SBP	12 weeks	Dapagliflozin significantly improved KCCQ-CS (+5.8 points), KCCQ-TS (+5.8), physical limitation scores (+5.3), KCCQ-OS (+4.5), 6MWT (+20.1m), and reduced weight (−0.72 kg). No significant difference in NT-proBNP or other biomarkers. Tolerability was comparable to placebo.
Filippatos et al., 2022 [11]	Randomized Controlled Trial	5988 HFpEF patients (LVEF >40%, NYHA II–IV); 49% with diabetes	Empagliflozin 10 mg daily	Placebo + usual therapy	Composite: 1st HF hospitalization or CV death; total HF hospitalizations; eGFR decline; hypoglycemic events	~26 months (median)	Empagliflozin significantly reduced the composite outcome in both diabetic and non-diabetic patients (HR ~0.78–0.79); renal protection; no hypoglycemia risk.
Peikert et al., 2022 [12]	Randomized Controlled Trial	6263 HFpEF patients (LVEF >40%, NYHA II–IV); mean age 71.7 ± 9.6 years; age groups <55 to ≥75	Dapagliflozin 10 mg daily	Placebo + standard care	Composite: CV death or worsening HF events; individual components; adverse events by age	Median 2.3 years	Dapagliflozin significantly reduced the composite risk of CV death or HF worsening across all age groups with consistent safety, including ≥75 years.
Packer et al., 2021 [13]	Randomized Controlled Trial	5988 HFpEF patients (LVEF >40%, NYHA II–IV); mean age not specified	Empagliflozin 10 mg daily	Placebo + standard care	Composite of CV death, HF hospitalization, urgent HF visits; intensive care need; diuretic escalation; NYHA class improvement	Median 26 months	Empagliflozin significantly reduced CV death or HF hospitalization (HR 0.77), improved NYHA class, and reduced outpatient and inpatient worsening HF events.
Butler et al., 2022 [14]	Randomized Controlled Trial	HFpEF patients with varying baseline health-related QoL (KCCQ-CSS tertiles <62.5, 62.5–83.3, ≥83.3); N not specified here	Empagliflozin 10 mg daily	Placebo + standard care	KCCQ-CSS, Total Symptom Score, Overall Summary Score, CV death or HF hospitalization, HF hospitalization rate	52 weeks	Empagliflozin improved QoL scores (KCCQ) at 12, 32, and 52 weeks; significantly reduced odds of deterioration and increased odds of ≥5/10/15 point improvements; consistent reduction in HF outcomes across all QoL strata.
Borlaug et al., 2023 [15]	Randomized Controlled Trial	38 HFpEF patients (NYHA class II–III, EF ≥50%) with elevated PCWP during exercise; median age 68; 66% women; 71% obese	Dapagliflozin 10 mg daily	Placebo	Change in PCWP at rest and during exercise, body weight, plasma volume, oxygen consumption, arterial lactate	24 weeks	Dapagliflozin significantly reduced PCWP at rest (-3.5 mm Hg) and during peak exercise (-5.7 mm Hg); also reduced body weight and plasma volume. No effect on red blood cell volume or oxygen consumption. Lower arterial lactate at submaximal exercise level.
Tromp et al., 2024 [16]	Randomized Controlled Trial	530 patients hospitalized for acute heart failure (de novo or decompensated), including 115 (22%) with HFpEF (EF ≥50%)	Empagliflozin 10 mg daily	Placebo	Hierarchical outcome of all-cause death, worsening heart failure events, and quality of life (KCCQ-TSS); safety	90 days	Empagliflozin showed consistent benefit across LVEF categories including HFpEF (win ratio 1.40; CI 0.87–2.23); improved symptoms and reduced events; favorable safety profile. Supported early in-hospital initiation regardless of EF.
Böhm et al., 2022 [17]	Randomized Controlled Trial	5,988 patients with HFpEF (LVEF ≥50%), stratified into age groups: <65, 65–74, 75–79, ≥80 years	Empagliflozin 10 mg daily	Placebo	Primary outcome: CV death or HF hospitalization; Total HF hospitalizations; eGFR decline; KCCQ-CSS; Adverse events	52 weeks	Empagliflozin significantly reduced the risk of CV death or HF hospitalization across all age groups, including ≥80 years; also reduced first and recurrent HFH, slowed eGFR decline, and improved quality of life. Efficacy and safety were consistent across age subgroups.
Butt et al., 2023 [18]	Randomized Controlled Trial	6,261 patients with HFmrEF/HFpEF (LVEF >40%), including 694 (11.1%) with mild-to-moderate COPD (severe COPD excluded)	Dapagliflozin 10 mg daily	Placebo	CV death or worsening HF; KCCQ-CSS; hospitalization rates; safety and tolerability in COPD vs. non-COPD patients	8 months (KCCQ), median trial duration ~2.3 years	Dapagliflozin reduced the risk of CV death or HF worsening similarly in patients with and without COPD. Improvements in KCCQ scores and hospitalization outcomes were consistent across both groups. No increase in adverse events or discontinuation due to COPD status.
Yang et al., 2024 [19]	Randomized Controlled Trial	Pooled patient-level data from DAPA-HF (HFrEF) and DELIVER (HFmrEF/HFpEF); wide EF spectrum; EQ-5D-5L assessed	Dapagliflozin 10 mg daily	Placebo	EQ-5D-5L VAS and index scores; clinical outcomes (CV death or worsening HF); health status correlation	Median ~2 years	Higher baseline EQ-5D-5L scores were associated with fewer comorbidities and better clinical status. Dapagliflozin significantly improved EQ-5D-5L VAS and index scores across all EF groups. Patients with better EQ-5D-5L scores had substantially lower risk of CV death or HF worsening.

Risk of Bias Assessment

The risk of bias assessment summarized in Table 2 indicates that the majority of the included randomized controlled trials were of high methodological quality, with a low risk of bias across all domains. Most studies demonstrated robust randomization processes, minimal deviations from intended interventions, complete outcome data, accurate outcome measurements, and appropriate reporting of results. Only one study raised some concerns regarding the randomization process, leading to an overall risk of bias judgment of "some concerns"; however, all other domains in that study were still rated as low risk. Overall, the evidence base appears reliable and methodologically sound.

**Table 2:** The risk of bias assessment of the included studies in the review. RCT – Randomized Controlled Trial

Author(s)	Study Design	Randomization Process	Deviations from Intended Interventions	Missing Outcome Data	Measurement of the Outcome	Selection of Reported Result	Overall Risk of Bias
Nassif et al., 2021 [10]	Multicenter RCT	Low Risk	Low Risk	Low Risk	Low Risk	Low Risk	Low Risk
Filippatos et al., 2022 [11]	RCT	Low Risk	Low Risk	Low Risk	Low Risk	Low Risk	Low Risk
Peikert et al., 2022 [12]	RCT	Low Risk	Low Risk	Low Risk	Low Risk	Low Risk	Low Risk
Packer et al., 2021 [13]	RCT	Low Risk	Low Risk	Low Risk	Low Risk	Low Risk	Low Risk
Butler et al., 2022 [14]	RCT	Low Risk	Low Risk	Low Risk	Low Risk	Low Risk	Low Risk
Borlaug et al., 2023 [15]	RCT	Some Concerns	Low Risk	Low Risk	Low Risk	Low Risk	Some Concerns
Tromp et al., 2024 [16]	RCT	Low Risk	Low Risk	Low Risk	Low Risk	Low Risk	Low Risk
Böhm et al., 2022 [17]	RCT	Low Risk	Low Risk	Low Risk	Low Risk	Low Risk	Low Risk
Butt et al., 2023 [18]	RCT	Low Risk	Low Risk	Low Risk	Low Risk	Low Risk	Low Risk
Yang et al., 2024 [19]	RCT (Pooled Data)	Low Risk	Low Risk	Low Risk	Low Risk	Low Risk	Low Risk

Discussion

Summary of Principal Findings

This systematic review synthesizes evidence from 10 randomized controlled trials investigating the role of SGLT2 inhibitors - namely empagliflozin and dapagliflozin - in patients with heart failure with preserved ejection fraction (HFpEF). The findings consistently indicate that these agents improve functional outcomes and reduce hospitalization risk. Trials such as Nassif et al. [10] demonstrated significant gains in KCCQ scores and six-minute walk test distances, reflecting enhanced quality of life and physical performance. Large-scale studies, including those by Filippatos et al. [11] and Packer et al. [13], reported reductions in the composite endpoint of cardiovascular death or heart failure hospitalization, with empagliflozin showing a hazard ratio (HR) of 0.79 (95% CI, 0.69-0.90) and dapagliflozin showing a HR of 0.82 (95% CI, 0.73-0.92). While hospitalization benefits were consistent and robust, the mortality signal remains less conclusive and warrants further long-term study.

These benefits were consistently observed across various patient subgroups, including older adults, those with comorbidities like diabetes and COPD, and in both ambulatory and hospitalized settings. However, important evidence gaps persist, particularly regarding sex- and race-specific responses, as women and minority populations remain underrepresented in major trials. Importantly, the safety profiles of SGLT2 inhibitors remained favorable, with minimal risk of hypoglycemia or adverse renal events. These findings align with the most recent recommendations from the American Heart Association (AHA) guidelines, which endorse SGLT2 inhibitors as a central therapeutic option for HFpEF management [20].

Comparison with Existing Literature

These findings build upon and strengthen the evolving evidence base that positions SGLT2 inhibitors as a transformative class of drugs in heart failure care, particularly for the HFpEF population, which has historically lacked effective treatment options [21]. Previous pharmacological strategies, such as RAAS inhibitors and beta-blockers, have shown limited or inconsistent impact on hospitalization rates or symptom burden in HFpEF patients [22]. In contrast, the consistent improvements in both patient-reported outcomes and clinical events reported in this review are aligned with recent recommendations by the European Society of Cardiology (ESC) and American Heart Association (AHA), which now endorse SGLT2 inhibitors for HFpEF management [20]. Moreover, trials like those by Butler et al. [14] and Yang et al. [19] offer new insights by demonstrating improvements in quality of life metrics across diverse baseline functional statuses, thereby expanding the applicability of these agents. These findings not only confirm the therapeutic value of SGLT2 inhibitors but also provide stronger grounds for their broader integration into HFpEF treatment guidelines.

Key Syntheses and Patterns

A notable pattern across the included trials is the consistency of benefit associated with SGLT2 inhibitors, irrespective of patient demographics or baseline clinical status. Trials such as those by Böhm et al. [17] and Peikert et al. [12] demonstrated that patients ≥75 years of age experienced similar reductions in heart failure hospitalizations as their younger counterparts, indicating age-independent efficacy. Likewise, the findings from Butler et al. [14] showed improvements in KCCQ scores across all tertiles of baseline health-related quality of life, suggesting that even patients with severely impaired functional status at baseline can derive significant symptomatic relief. The beneficial effects of SGLT2 inhibitors were also preserved across comorbid groups, including those with diabetes (Filippatos et al. [11]) and mild-to-moderate COPD (Butt et al. [18]). While most studies used a standard daily dose of 10 mg, variations in treatment duration - from 12 weeks in Nassif et al. [10] to over two years in Filippatos et al. [11] - still yielded clinically relevant improvements, reinforcing the early and sustained efficacy of these agents.

Clinical Interpretation

The clinical benefits observed with SGLT2 inhibitors in HFpEF patients may be explained by multiple interrelated physiological mechanisms. Unlike traditional heart failure therapies that rely on neurohormonal modulation, SGLT2 inhibitors primarily act through metabolic and hemodynamic pathways [23]. Borlaug et al. [15] provided direct evidence of reduced pulmonary capillary wedge pressure (PCWP) both at rest and during exercise with dapagliflozin, implying a lowering of cardiac preload and left-sided filling pressures. These hemodynamic changes are likely mediated by mild osmotic diuresis and natriuresis, leading to reduced plasma volume and interstitial fluid overload. Additional mechanisms may include improvements in vascular endothelial function, reductions in systemic inflammation and myocardial fibrosis, and enhanced myocardial energetics. Weight loss and reduced arterial stiffness, as observed in several trials, further contribute to the alleviation of HFpEF symptoms, particularly exercise intolerance and dyspnea.

Heterogeneity and Limitations

While the overall findings across trials are compelling, several sources of heterogeneity and limitations should be acknowledged. There were variations in ejection fraction cutoffs, with some studies including patients with LVEF >40% (Filippatos, Peikert [11,12]), while others specifically focused on EF ≥50% (Borlaug, Böhm [15,17]), potentially affecting the generalizability of results. Patient populations also differed in terms of NYHA class distribution, baseline QoL, and comorbidity burden. Additionally, outcome measures were not uniform; while most studies used KCCQ as the primary functional outcome, Yang et al. [19] utilized EQ-5D-5L, and not all trials assessed exercise tolerance or hemodynamics. Trial duration varied significantly - from 12 weeks in Nassif et al. [10] to 2.5 years in Peikert et al. [12] - introducing variability in long-term outcome interpretation. Lastly, studies like that of Borlaug et al. [15] had small sample sizes, limiting statistical power, while others included post-hoc subgroup analyses, which may introduce selection or analytical biases.

Clinical Implications

The consistent improvements observed in both patient-reported and clinical outcomes affirm the utility of SGLT2 inhibitors as a central component of HFpEF management. Unlike traditional therapies that have largely failed to improve symptom burden or hospitalization rates, empagliflozin and dapagliflozin offer tangible benefits that are both meaningful to patients and supported by robust trial data [24,25]. These findings suggest that SGLT2 inhibitors should be considered as first-line agents for symptomatic HFpEF patients, especially those who experience frequent exacerbations or diminished functional capacity. Furthermore, their additional advantages in lowering body weight, improving glycemic control, and preserving renal function make them particularly valuable in patients with overlapping conditions such as type 2 diabetes, obesity, or early-stage chronic kidney disease - common comorbidities in the HFpEF population.

Gaps and Future Directions

Despite the promising results, important questions remain regarding the long-term and comparative effectiveness of SGLT2 inhibitors in HFpEF. Most trials assessed intermediate outcomes such as symptom improvement and HF hospitalization, while long-term effects on all-cause and cardiovascular mortality are still under investigation. There is also a lack of head-to-head comparisons between SGLT2 inhibitors and other emerging treatments such as mineralocorticoid receptor antagonists or neprilysin inhibitors in HFpEF [26]. Moreover, data on real-world effectiveness, especially in low- and middle-income countries (LMICs), remain sparse. Future research should aim to identify biomarkers that predict response to therapy, examine cost-effectiveness in diverse healthcare systems, and explore combination therapies that may offer synergistic benefits. Expanding representation of underdiagnosed and under-treated populations, such as women and the elderly with preserved EF, is also essential for refining treatment strategies.

## Conclusions

This review consolidates robust evidence supporting SGLT2 inhibitors as the first effective pharmacologic option to improve functional status and reduce hospitalizations in HFpEF - a condition long characterized by therapeutic inertia. Through consistent benefits observed across age groups, comorbid conditions, and baseline functional capacities, empagliflozin and dapagliflozin have emerged as foundational therapies that address both the symptomatic and clinical burden of HFpEF. Their favorable safety profiles and pleiotropic benefits further support their integration into routine clinical practice, marking a critical shift toward personalized and evidence-based care in this challenging patient population.
